# Prevention, recognition, and management of adverse events associated with gemtuzumab ozogamicin use in acute myeloid leukemia

**DOI:** 10.1186/s13045-020-00975-2

**Published:** 2020-10-15

**Authors:** Jorge E. Cortes, Marcos de Lima, Hervé Dombret, Elihu H. Estey, Sergio A. Giralt, Pau Montesinos, Christoph Röllig, Adriano Venditti, Eunice S. Wang

**Affiliations:** 1 Georgia Cancer Center, 1410 Laney Walker Road, CN2116, Augusta, GA 30912 USA; 2 University Hospitals Seidman Cancer Center, Cleveland, OH USA; 3 Hôpital Saint-Louis, Université Paris, Paris, France; 4 University of Washington, Seattle, WA USA; 5 Memorial Sloan Kettering Cancer Center, New York, NY USA; 6 Hospital Universitari I Politècnic La Fe, Valencia, Spain; 7 CIBERONC, Instituto de Salud Carlos III, Madrid, Spain; 8 University Hospital Carl Gustav Carus, Dresden, Germany; 9 Tor Vergata University of Rome, Rome, Italy; 10 Roswell Park Comprehensive Cancer Center, Buffalo, NY USA

**Keywords:** Acute myeloid leukemia, Adverse events, Gemtuzumab ozogamicin, Treatment management, VOD/SOS

## Abstract

Gemtuzumab ozogamicin (GO), a humanized anti-CD33 monoclonal antibody conjugated to the cytotoxic antibiotic agent calicheamicin, is approved for the treatment of newly-diagnosed CD33 + AML in adults and children ≥ 1 month old, and relapsed or refractory CD33 + AML in adults and children ≥ 2 years old. GO treatment has been associated with an increased risk of hepatotoxicity and hepatic veno-occlusive disease/sinusoidal obstruction syndrome (VOD/SOS), especially following hematopoietic stem cell transplantation. Other non-specific serious adverse events (SAEs) associated with GO treatment are myelosuppression, bleeding/thrombocytopenia, infusion-related reaction, and tumor lysis syndrome. This report summarizes an expert panel of physicians’ recommendations for the evaluation and management of SAEs following GO, emphasizing the prevention and management of VOD/SOS.

## Background

Gemtuzumab ozogamicin (GO) is an antibody drug conjugate comprising a CD33-directed monoclonal antibody covalently linked to the cytotoxic agent N-acetyl gamma calicheamicin. The CD33 antigen is expressed on the vast majority of myeloid leukemic blasts and immature normal cells of myelomonocytic lineage, but not on normal hematopoietic stem cells [1–3]. GO was granted accelerated approval by the United States (US) Food and Drug Administration (FDA) in 2000 as monotherapy (9 mg/m^2^ repeated after 14 days on approximately 28-day cycles) for patients ≥ 60 years of age with CD33-positive (CD33 +) acute myeloid leukemia (AML) in first relapse and who were not candidates for standard chemotherapy [4]. Approval was granted based on three phase II studies in which the administration of single-agent GO (9 mg/m^2^ on days 1 and 14 of induction) resulted in a 26% rate of complete remission with (CR) and without (CRp) platelet recovery in patients > 60 years of age. Myelosuppression and hepatotoxicity were the most serious toxicities reported in these early clinical trials.

GO was voluntarily withdrawn from the market in 2010 after a post-approval trial (Southwest Oncology Group [SWOG] S0106) failed to confirm benefit in patients randomized to GO (6 mg/m^2^ on day 4) plus daunorubicin (DNR, 45 mg/m^2^ per day on days 1, 2, and 3) and cytarabine (AraC; 100 mg/m^2^ per day by continuous infusion on days 1–7) versus DNR (60 mg/m^2^) plus AraC alone [5]. The SWOG S0106 study was terminated early by the data monitoring committee due to a higher incidence of fatal induction toxicities in the GO arm, including infection and/or febrile neutropenia, central nervous system hemorrhage, acute respiratory distress syndrome/dyspnea, lung hemorrhage, and liver dysfunction. However, several other phase II and III studies were underway exploring alternative doses and schedules of GO in various settings. Results of several of these studies suggested a possible benefit and a more acceptable safety profile of GO, leading to questions on whether the optimal dose schedule of GO had been investigated [6–11].

The pivotal phase III ALFA-0701 study examined the efficacy and safety of smaller, fractionated doses of GO administered in combination with standard chemotherapy in patients with previously untreated AML [6]. Patients were given an induction course of DNR (60 mg/m^2^ IV, days 1–3) and AraC (200 mg/m^2^ IV continuous infusion for 7 days) with or without GO (3 mg/m^2^ IV infusion on days 1, 4, and 7) after premedication with methylprednisolone. In patients with > 10% persistent leukemic blasts following bone marrow aspiration, a second induction course was given with DNR (60 mg/m^2^ IV per day for 2 days) and AraC (1000 mg/m^2^ per 12 h, IV infused over 2 h for 3 days) without GO, followed by daily lenograstim until neutrophil recovery. Patients in CR or CRp received two consolidation courses of DNR (60 mg/m^2^ IV for 1 day [first course] or 2 days [second course]) plus AraC (1000 mg/m^2^ per 12 h, IV infused over 2 h on days 1–4), with or without GO (3 mg/m^2^ IV on day 1) [6]. Compared with standard chemotherapy alone, the addition of GO was associated with improved event-free survival and relapse-free survival. Some severe adverse events (AEs) were more frequent in the GO arm, most notably delayed platelet recovery, persistent thrombocytopenia, and veno-occlusive disease/sinusoidal obstruction syndrome (VOD/SOS) [6, 12]. Based on ALFA-0701 [6] and other clinical trials [7–11], in conjunction with pharmacokinetic/pharmacodynamic modeling to validate the safety and efficacy of the fractionated dosing regimens [13], GO was reapproved by the US FDA in 2017 for the treatment, in combination with standard chemotherapy, of newly-diagnosed CD33 + AML in adults, and as a single agent for relapsed or refractory (R/R) CD33 + AML in adults and pediatric patients ≥ 2 years old [14]. In June 2020, the FDA label was extended to also include GO in combination with standard chemotherapy for newly-diagnosed pediatric patients ≥ 1 month old [15]. GO was also approved by the European Medicines Agency (EMA) in February 2018 in combination therapy with DNR and AraC for the treatment of patients ≥ 15 years old with previously untreated, de novo CD33 + AML, except acute promyelocytic leukemia (APL) [16].

Increased clinical experience with GO, and refinements in GO dosing and administration strategies, have resulted in a decrease in the frequency and/or severity of some of the toxicities observed in early clinical trials. However, safety concerns with GO that require adequate and prompt recognition and management still exist. An increased risk of hepatic VOD/SOS has been linked to GO use, especially after hematopoietic stem cell transplantation (HSCT). Other serious AEs include myelosuppression, infusion-related reaction, and tumor lysis syndrome (TLS). Here, we present a review of some of the most serious AEs associated with GO, with emphasis on the prevention and management of VOD/SOS.

A plain language summary of this article is available in Supplementary Information (see Additional file 1).

### Veno-occlusive disease/sinusoidal obstruction syndrome

VOD/SOS is a serious and potentially life-threatening complication that is primarily observed after HSCT and results from damage to the sinusoidal endothelium caused by toxic metabolites that are generated during the conditioning regimen [17, 18]. Diagnosis of VOD/SOS is based on weight gain, ascites, tender hepatomegaly, and jaundice (Table 1). VOD/SOS most commonly develops within 30 days after transplantation, but may occur later [19, 20]. Risk factors for the development of the complication include, but are not limited to, myeloablative conditioning regimens, very young or old age, advanced disease at transplantation, and a Karnofsky score < 90 [21].

**Table 1:** EBMT criteria for SOS/VOD in adults and children

Adults [38]	Children [39]
*Classical (first 21 days after HSCT)* Bilirubin ≥ 2 mg/dL and 2 of the following criteria must be present: • Painful hepatomegaly • Weight gain > 5% • Ascites *Late onset (*> *21 days after HSCT)* Classical VOD/SOS beyond day 21 OR Histologically proven SOS/VOD OR ≥ 2 of the following criteria must be present: • Bilirubin ≥ 2 mg/dL (or 34 μmol/L) • Painful hepatomegaly • Weight gain > 5% • Ascites AND hemodynamical and/or ultrasound evidence of SOS/VOD	No limitation for time of onset of SOS/VOD The presence of ≥ 2 of the following:^a^ • Unexplained consumptive and transfusion-refractory thrombocytopenia^b^ • Otherwise unexplained weight gain on 3 consecutive days despite the use of diuretics or a weight gain > 5% above baseline value • Hepatomegaly^c^ (best if confirmed by imaging) above baseline value • Ascites^c^ (best if confirmed by imaging) above baseline value • Rising bilirubin from a baseline value on 3 consecutive days or bilirubin ≥ 2 mg/dL within 72 h

*EBMT* European Society for Blood and Marrow Transplantation; *HSCT* hematopoietic stem cell transplantation; *SOS/VOD* sinusoidal obstruction syndrome/veno-occlusive disease

^a^With the exclusion of other potential differential diagnoses

^b^≥ 1 weight-adjusted platelet substitution/day to maintain institutional transfusion guidelines

^c^Suggested: imaging immediately before HSCT to determine baseline value for both hepatomegaly and ascites

GO therapy has been linked to the development of VOD/SOS, both in patients who also receive a HSCT and those who do not [22]. The pathophysiology of GO-associated VOD/SOS is not fully understood but may result from the delivery of calicheamicin to CD33 + sinusoidal endothelial cells, as such cells are known to express this surface protein [23]. Early clinical trials in patients with AML reported VOD/SOS rates of 3% with ≤ 6 mg/m^2^ GO as monotherapy or in combination with non-hepatotoxic agents, 28% if GO was administered with thioguanine, and 15% with 9 mg/m^2^ GO as monotherapy [22]. Furthermore, observational studies reported VOD/SOS rates of 15–40% if an HSCT was performed within 3 months of GO administration [22]. For those who did not proceed to HSCT, a VOD/SOS incidence of 9% was reported, occurring at a median of 10 days following administration of GO [22]. In the ALFA-0701 trial, 4.6% (*n* = 6/131) of patients in the GO arm and 1.5% (n = 2/137) of patients in the control arm developed VOD/SOS [12]. In a retrospective analysis of the subgroup of patients who received HSCT on study (GO: *n* = 32; control: *n* = 53), VOD/SOS after HSCT or during conditioning occurred in 2 patients in the GO arm and 1 patient in the control arm who received GO after HSCT; all but 1 patient in the GO arm recovered. Importantly, an interval of ≥ 3 months was recommended between last GO dose and HSCT as a means to reduce the risk of VOD/SOS. The panel noted, however, that the ALFA-0701 trial used a 2-month threshold instead. The development of VOD/SOS was not associated with a significant difference in overall survival after HSCT between treatment arms [12, 24]. In a phase I/II study, GO (2 mg/m^2^) as part of a preparative regimen for allogeneic HSCT was considered safe in patients with high-risk CD33 + AML, CML, or myelodysplastic leukemias, with 1 case of VOD/SOS among 52 patients [25].

In an expanded access protocol study, which allowed compassionate use of GO in patients with R/R AML or acute promyelocytic leukemia, 322 patients with R/R AML were treated with single-agent GO ≥ 9 mg/m^2^ or GO in combination with anthracyclines and/or nucleoside analog-containing regimens, or hypomethylating agents. Hepatotoxicity was reported in 5 patients: VOD and drug-induced liver injury (*n* = 1 each; 0.7%) in patients treated with monotherapy for AML; and VOD (*n* = 3; 1.6%; 1 fatal event) in patients treated with combination therapy [26]. In a separate retrospective registry analysis of adults with AML who underwent first myeloablative allogeneic HSCT between 2008 and 2011, 137 patients treated with GO and 548 matched controls with no GO exposure were assessed for VOD. The overall incidence of VOD was low in both cohorts (4% each), with a similar survival probability at 1 year (GO: 53%; control: 57%). Severe VOD was observed in 3% and 1% of patients in the GO and no GO cohorts, respectively [27].

Based on these recently published data, the risk of VOD/SOS with GO therapy, especially in patients who proceed to HSCT, appears to be lower than originally reported [22]. The decrease in incidence may reflect the use of smaller and/or fractionated GO dosing, but other factors such as improved knowledge and management of VOD/SOS risk factors, advancements in preventative care, and treatment may also play a role [28, 29]. Nevertheless, VOD/SOS is a potentially life-threatening complication, and patients receiving GO should continue to be monitored closely for the development of the disease. Early recognition and prompt intervention may make a significant difference to the outcome of patients with VOD/SOS.

#### Prevention of VOD/SOS

Based on the increased risk of VOD/SOS with GO treatment, patients with clinically significant prior hepatic impairment (defined as prior history of hepatitis, portal hypertension, cirrhosis, or other chronic liver diseases) or baseline hepatic impairment (defined by elevated liver function tests [LFTs]) should not be given GO unless the potential benefits are considered to outweigh the potential risks (Table 2). In addition, for patients with R/R AML who have received prior HSCT (particularly recently) the risk–benefit ratio of GO treatment should be carefully evaluated [30].

**Table 2:** Summary of recommendations for prevention and monitoring of VOD/SOS

Prevention	Monitoring
Avoid GO in patients with significant prior or baseline hepatic impairment or patients with R/R AML with prior HSCT For patients proceeding to HSCT, an interval of ≥ 3 months between HSCT and last GO dose is recommended and treatment plans should be communicated to the transplant team at the outset The risk of hepatic damage due to concomitant medications should be assessed to avoid harmful drug interactions Prophylactic ursodeoxycholic acid is recommended	All patients receiving GO should be closely monitored for signs and symptoms of VOD/SOS (e.g., weight gain; see Table 1), especially in those receiving azoles during GO treatment LFTs should be monitored prior to each GO dose Close monitoring of LFTs is recommended in the post-transplant period for patients proceeding to HSCT GO dose should be postponed in patients with total bilirubin > 2 × ULN and AST and/or ALT > 2.5 × ULN until recovery of total bilirubin to ≤ 2 × ULN and AST/ALT to ≤ 2.5 × ULN prior to each dose Consider omitting the scheduled GO dose if the delay between sequential infusions is > 2 days

*ALT* alanine aminotransferase; *AST* aspartate aminotransferase; *GO* gemtuzumab ozogamicin; *HSCT* hematopoietic stem cell transplantation; *LFTs* liver function tests; *ULN* upper limit of normal; *VOD/SOS* veno-occlusive disease/sinusoidal obstruction syndrome

The use of GO in patients with favorable-risk cytogenetics is recommended. These patients have a low risk of VOD/SOS as they usually will not undergo an allogeneic HSCT and have shown significantly improved overall survival with the addition of GO to chemotherapy vs chemotherapy alone (OS HR [95% CI] 0.32 [0.18–0.59]) [10]. This benefit was less pronounced among those with intermediate cytogenetics (0.86 [0.70–1.07]), with no survival benefit reported for those with adverse cytogenetics (1.17 [0.82–1.68]). For patients proceeding to HSCT, a delay of ≥ 3 months following the last dose of GO should be considered. However, prospective studies are needed to better characterize the optimal timing between HSCT and last GO dose and the impact of this variable on VOD/SOS risk (Table 2). Where possible, we recommend prophylactic ursodeoxycholic acid is given to all patients treated with GO in induction and at high risk of VOD, and in patients treated with GO prior to HSCT. Ursodeoxycholic acid, a hydrophilic bile acid and antioxidant, reduces hydrophobic bile acids that may be toxic to hepatic parenchymal cells and has been linked with a reduction in the incidence of hepatic VOD/SOS, albeit with low quality of evidence [31]. The benefit of ursodeoxycholic acid in the context of GO treatment remains to be established. Finally, it is recommended that the risk of hepatic damage due to concomitant medication should be carefully assessed to avoid any potentially harmful drug interactions.

#### Monitoring VOD/SOS

All patients receiving GO should be closely monitored for signs and symptoms of VOD/SOS, including hepatomegaly, weight gain, and ascites. LFTs should be monitored prior to each dose of GO (Table 2). Additionally, close monitoring of LFTs during the post-HSCT period is recommended for patients who proceed to HSCT. Treating physicians should also take special care in monitoring LFTs in patients receiving azoles during treatment with GO. However, before deciding to discontinue azoles, the risk of VOD/SOS should be considered alongside the risk of fungal infection in each patient. In patients who develop abnormal LFTs while on GO therapy, the frequency of monitoring LFTs and clinical signs and symptoms of hepatotoxicity should be increased. GO scheduled doses should be postponed in patients with total bilirubin > 2 × upper limit of normal (ULN) and aspartate aminotransferase (AST) and/or alanine transaminase (ALT) > 2.5 × ULN until recovery of total bilirubin to ≤ 2 × ULN and AST/ALT to ≤ 2.5 × ULN prior to each dose. Treating physicians may consider omitting the scheduled GO dose if it is delayed > 2 days between sequential infusions. Transient elastography (FibroScan®) is currently being investigated as a potential tool for monitoring the clinical manifestations of VOD/SOS (ClinicalTrials.gov, NCT03426358). Finally, increasing staff awareness of the risk of VOD/SOS is important for early identification of the disease and to facilitate prompt treatment with defibrotide.

#### Treatment of VOD/SOS

Treatment of VOD/SOS should be conducted according to guidelines from the British Committee for Standards in Haematology and the British Society for Blood and Marrow Transplantation, and in line with European Society for Blood and Marrow Transplantation (EBMT) recommendations [21, 32]. Briefly, treatment of VOD/SOS must be started as soon as possible, with fluid and sodium balance plus careful use of diuretics introduced at the first suspicion of the disease. While symptomatic measures are the first step in treatment, defibrotide is the only proven curative treatment for VOD/SOS and is the recommended approach in adults and children with VOD/SOS. A recently-published literature analysis supports the efficacy of defibrotide for post-HSCT VOD/SOS among patients who have previously received GO [33]. Defibrotide appears to protect endothelial cells and restore thrombotic-fibrinolytic balance. Early discussion with critical care specialists and a specialist hepatology unit is recommended, and treatment options including transjugular intrahepatic portosystemic shunt and hepatic transplantation may be considered.

### Myelosuppression

Myelosuppression is a common side effect of GO treatment [6]. In the ALFA-0701 trial, the duration of treatment-induced neutropenia was longer in the GO arm than the control arm after each consolidation course (median 13 vs. 10 days in consolidation 1, 15 vs. 13 days in consolidation 2), but not after induction (median 22 days for both treatment arms) [6].

#### Recommendations for Managing Neutropenia

Complete blood counts should be performed prior to each GO dose, and patients should be monitored for signs and symptoms of infection or other effects of myelosuppression during treatment (Table 3). Dose modification is not necessary prior to the first dose of treatment; however, dose adjustment or treatment discontinuation may be necessary in patients with persistent neutropenia. The decision to discontinue GO should be based on careful assessment of the benefits and risks of treatment and be considered within the context of the overall treatment strategy for individual patients.

**Table 3:** Summary of recommendations for managing other serious AEs with GO

AE	Recommendations
Thrombocytopenia	Monitor complete blood counts prior to each dose and perform routine clinical and laboratory surveillance testing during and after treatment Monitor for signs and symptoms of infection or bleeding/hemorrhage, or other effects of myelosuppression during treatment Manage severe bleeding, hemorrhage, or persistent thrombocytopenia using dose delay or permanent discontinuation of GO and provide supportive care per standard practice If platelet count recovers to ≥ 100,000/mm^3^ ≤ 14 days following the planned start date of the consolidation course, initiate treatment with GO If platelet count takes longer than 14 days to recover to ≥ 50,000/mm^3^, or if platelet count does not recover to ≥ 50,000/mm^3^, consolidation therapy should be re-evaluated, and a bone marrow aspirate should be performed to re-assess the patient’s status
Neutropenia	Monitor complete blood counts prior to each dose and perform routine clinical and laboratory surveillance testing during and after treatment Monitor for signs and symptoms of infection or other effects of myelosuppression during treatment Management of persistent neutropenia may require dose adjustment or treatment discontinuation
Infusion-related reactions	GO should not be administered in patients with hypersensitivity to the active substance or any excipients Pre-medication with a corticosteroid, antihistamine, and acetaminophen is recommended 1 h prior to GO dosing Infusion of GO should be performed under close clinical monitoring, including monitoring of pulse, blood pressure, and temperature Closely monitor signs and symptoms of infusion-related reactions (e.g., fever, chills, hypotension, tachycardia, respiratory symptoms) that may occur during the first 24 h after administration, until signs and symptoms completely resolve If infusion-related reactions occur, interrupt the infusion and institute appropriate medical management • For mild, moderate, or severe infusion-related reactions, consider resuming the infusion at no more than half the rate at which the reaction occurred when symptoms resolve • GO should be permanently discontinued upon occurrence of a severe infusion-related reaction or any life-threatening infusion-related reaction
Tumor lysis syndrome	In patients with hyperleukocytic AML, cytoreduction with leukapheresis, oral hydroxyurea, or cytarabine with/without hydroxyurea should be achieved prior to administration of GO When patients receive cytarabine for leukoreduction, the treating physician should consider modifying the induction dosing schedule • Monitor for signs and symptoms of TLS and treat according to standard medical practice • Appropriate measures should be taken to prevent the development of TLS-related hyperuricemia, such as hydration and administration of antihyperuricemics (e.g., allopurinol) or other agents for the treatment of hyperuricemia (e.g., rasburicase)

*AE* adverse event; *AML* acute myeloid leukemia; *GO* gemtuzumab ozogamicin; *TLS* tumor lysis syndrome

### Thrombocytopenia

In the ALFA-0701 trial, persistent grade 3/4 thrombocytopenia was observed in 16% of patients in the GO arm compared with 3% in the control arm. Notably, the term CRp was first used in relation to the use of GO, recognizing that approximately half of responders did not fully recover platelet counts [34]. The median number of platelet transfusion episodes was higher in the GO arm than the control arm after each treatment course [6].

#### Recommendations for Managing Thrombocytopenia

In patients receiving GO, monitoring of thrombocytopenia should be based on complete blood counts (at least prior to each dose but typically more frequently, e.g., 2–3 times per week) and routine clinical and laboratory surveillance testing (performed during and after treatment) (Table 3). Patients should be monitored for signs and symptoms of bleeding/hemorrhage. Dose delays or permanent discontinuation of GO, and supportive care per standard practice, are recommended to manage severe bleeding, hemorrhage, or persistent thrombocytopenia. In the ALFA-0701 trial, the study protocol (following an amendment on December 2009) recommended that GO should not be used during consolidation in patients with a platelet count < 100 × 10^9^ per L by day 45 after the initiation of chemotherapy [6].

### Infusion-related reactions

Monoclonal antibodies have the potential to induce infusion reactions [35]. Symptoms are often mild and treatable with supportive care. Signs and symptoms of infusion reactions may include fever, chills, hypotension, tachycardia, hypoxia, and respiratory failure [14]. Particular care should be taken during the first infusion as subsequent infusions are less prone to induce a reaction.

#### Recommendations for Managing Infusion-related Reactions

GO should not be administered in patients with known hypersensitivity to the active substance or any excipients. In patients receiving GO, pre-medication with a corticosteroid, antihistamine, and acetaminophen is recommended 1 h prior to dosing. Infusion of GO should be performed under close clinical monitoring, including monitoring of pulse, blood pressure, and temperature. If infusion-related reactions occur, interrupt the infusion and institute appropriate medical management (Table 3). GO should be permanently discontinued upon occurrence of a severe infusion-related reaction or any life-threatening infusion-related reaction.

### Tumor lysis syndrome

TLS can occur with any chemotherapeutic agent used in the treatment of leukemia, including GO [14]. TLS results in electrolyte and metabolic disturbances, which can lead to renal impairment, cardiac arrhythmias, seizures, and death [36].

#### Recommendations for Managing Tumor Lysis Syndrome

Patients receiving GO should be monitored for signs and symptoms of TLS and treated accordingly (Table 3). In patients with high white blood cell count (e.g. ≥ 30,000/µL [15]), cytoreduction with leukapheresis or administration of oral hydroxyurea or AraC should be utilized to bring the white cell count to below 30,000/µL, and ideally as close to normal as possible, prior to administration of GO. When patients receive AraC for leukoreduction, the treating physician may consider modifying the induction dosing schedule (Table 3). Appropriate measures should be taken to prevent the development of tumor lysis-related hyperuricemia, such as hydration and administration of antihyperuricemics (e.g., allopurinol) or other agents for the treatment of hyperuricemia (e.g., rasburicase), in patients at risk of developing TLS. A scoring system is available to assess the individual risk of patients developing TLS [37]. If TLS occurs despite these measures, prompt intervention is required with hydration and management of electrolyte abnormalities. Rasburicase is often effective in promptly decreasing uric acid and reducing risk of renal dysfunction.

## Conclusions

Gemtuzumab ozogamicin is approved for the treatment of newly-diagnosed CD33 + AML in adults, and for relapsed or refractory CD33 + AML in adults and pediatric patients ≥ 2 years old. However, treatment with GO has been associated with an increased risk of hepatic VOD/SOS (especially following hematopoietic stem cell transplantation) and other serious AEs of concern. Based on recently published data, the risk of VOD/SOS with GO therapy appears less than originally suggested, reflecting improved knowledge and management of VOD/SOS risk factors, advancements in preventative care and treatment, and the use of fractionated GO dosing. This report has summarized the recommendations of an expert panel of physicians for the evaluation and management of serious AEs associated with GO, with a focus on the prevention, mitigation, and management of VOD/SOS. While patients receiving GO should continue to be monitored closely for the development of VOD/SOS and other serious AEs, strategies for effective AE management to improve patient outcomes and quality of life are provided.

## Supplementary information

**Additional file 1.** Prevention, recognition, and management of adverse events associated with gemtuzumab ozogamicin use in acute myeloid leukemia.

## Data Availability

Not applicable.

## Abbreviations

AE: Adverse events
ALT: Alanine transaminase
AML: Acute myeloid leukemia
APL: Acute promyelocytic leukemia
AST: Aspartate aminotransferase
AraC: Cytarabine
CD33 +: CD33-positive
CR: Complete remission
CRp: Complete remission without platelet recovery
DNR: Daunorubicin
EMA: European Medicines Agency
FDA: Food and Drug Administration
GO: Gemtuzumab ozogamicin
HSCT: Hematopoietic stem cell transplantation
LFT: Liver function tests
SOS: Sinusoidal obstruction syndrome
SWOG: Southwest Oncology Group
R/R: Relapsed or refractory
TLS: Tumor lysis syndrome
ULN: Upper limit of normal
US: United States
VOD: Veno-occlusive disease
