# Supplementary material for: Prevention, recognition, and management of adverse events associated with gemtuzumab ozogamicin use in acute myeloid leukemia
Source: J Hematol Oncol. 2020 Oct 15;13:137. doi: 10.1186/s13045-020-00975-2 (PMC7559451; doi:10.1186/s13045-020-00975-2)
Supplement: Supplementary file 1 — Additional file 1. Prevention, recognition, and management of adverse events associated with gemtuzumab ozogamicin use in acute myeloid leukemia. [file 13045_2020_975_MOESM1_ESM.pdf]

Please note that this summary only contains information from the full scientific article:
View Scientific Article

Preventing and managing side effects of gemtuzumab ozogamicin for people with acute myeloid leukemia

Date of summary: June 2020

The full title of this article: Prevention, recognition and management of adverse events associated with gemtuzumab ozogamicin use in acute myeloid leukemia

The purpose of this plain language summary is to help you to understand the findings from recent research.

Gemtuzumab ozogamicin is approved to treat the condition under study that is discussed in this summary.

Researchers must look at the results of many types of studies to understand whether a study drug works, how it works, and whether it is safe to prescribe to patients.

This summary reports information from a review article. The findings might be different from other review articles.

More information can be found in the scientific article of this analysis, which you can access here: View Scientific Article

Acute myeloid leukemia
<uh-KYOOT MY-eh-loyd loo-KEE-mee-uh>

Gemtuzumab ozogamicin
<gem-TOO-zoo-mab OH-zoh-ga-MIH-sin>

Neutrophil <NOO-troh-fil>

Sinusoidal obstruction syndrome
<SINE-yoo-SOY-dul ub-STRUK-shun SIN-drome>

Tumor lysis syndrome
<TOO-mer LY-sis SIN-drome>

Veno-occlusive <VEE-noh-uh-KLOO-siv>

What does this summary look at?

- This summary describes some recommendations from a group of doctors who were part of an expert panel.
  - These doctors had been involved in treating people with acute myeloid leukemia (AML for short) who received gemtuzumab ozogamicin plus standard chemotherapy.
- AML is a type of blood cancer where the body produces too many of a certain type of white blood cell that does not develop properly.
  - This can prevent the body from producing normal blood cells.
- Gemtuzumab ozogamicin is a treatment for people with AML, and is given through a drip into a vein.
  - Gemtuzumab ozogamicin can be combined with standard chemotherapy because the combination may make chemotherapy work better.
  - Gemtuzumab ozogamicin works by finding and destroying AML cancer cells that have a protein called CD33 on their surface.
- The expert panel discussed:
  - Side effects* of gemtuzumab ozogamicin when given alone or in combination with standard chemotherapy
  - How doctors can prevent and manage these side effects.
- This summary focuses on specific side effects that can be serious** and that some people have experienced when receiving gemtuzumab ozogamicin:
  - A condition where blood vessels in the liver become blocked. This is called veno-occlusive disease/sinusoidal obstruction syndrome (VOD/SOS for short)
  - Low levels of healthy blood cells
  - Allergic reactions
  - A condition called tumor lysis syndrome. In tumor lysis syndrome, cancer cells split open and spill their contents into the blood. This changes the levels of different chemicals in the blood.

*A side effect is something (expected or unexpected) that you feel was caused by a medicine or treatment you take.

**A side effect is considered "serious" when it is life-threatening, needs hospital care, or causes lasting problems.

Who took part in the expert panel?

- The expert panel involved 9 doctors from different hospitals and research centers around the world.
  - They had experience in treating people with AML who received gemtuzumab ozogamicin plus standard chemotherapy.

What were the main recommendations from the expert panel?

VOD/SOS: Where blood vessels in the liver become blocked

- The expert panel focused mostly on VOD/SOS, because this possible side effect* can be very serious.
  - VOD/SOS is more common when a person receives a bone marrow transplant after gemtuzumab ozogamicin treatment, particularly if within a few months.
- Rates of VOD/SOS among people receiving gemtuzumab ozogamicin are lower now than when gemtuzumab ozogamicin was first used many years ago. This could be due to doctors:
  - Prescribing lower doses of gemtuzumab ozogamicin than they used to
  - Giving multiple small doses of gemtuzumab ozogamicin, instead of a few larger doses
  - Having access to greater knowledge and more advanced methods for preventing and treating VOD/SOS.
- To help prevent VOD/SOS, doctors should:
  - Avoid giving gemtuzumab ozogamicin to people who:
    - Have known liver problems
    - Have had their AML return after a previous bone marrow transplant, unless the benefits are found to outweigh the risks
  - Wait at least 3 months after the last dose of gemtuzumab ozogamicin before giving the person a bone marrow transplant
  - Provide medications that may help prevent VOD/SOS, and avoid medications that could cause liver problems.
- To monitor for VOD/SOS, doctors should:
  - Do blood tests to make sure that the person's liver is working properly before each dose of gemtuzumab ozogamicin, and also after receiving a bone marrow transplant
    - Doctors should delay gemtuzumab ozogamicin treatment if blood tests show abnormal levels of substances produced by the liver
  - Check people for symptoms, such as pain in the liver area and yellowing of the skin (jaundice).
- To treat VOD/SOS, options include:
  - Using a treatment called defibrotide as soon as possible
  - Ensuring people have balanced levels of fluids and electrolytes.

Low levels of healthy blood cells

- Gemtuzumab ozogamicin often affects the ability of the bone marrow to produce healthy blood cells. People may have low levels of:
  - A certain type of white blood cell called a neutrophil
  - Cells that help the blood to clot, called platelets; they may need to receive platelet transfusions.
- To help manage these side effects, doctors should:
  - Test blood cell levels during and after treatment
  - Monitor people for signs and symptoms such as infection or bleeding
  - Reduce the dose or stop using gemtuzumab ozogamicin, for people with low levels of neutrophils that do not recover within a reasonable time
  - Delay or stop using gemtuzumab ozogamicin and use supportive care,*** for people with low platelet levels that do not improve or who have severe bleeding.

Allergic reactions

- Allergic reactions can happen while people are receiving infusions of gemtuzumab ozogamicin. They are usually mild.
- To prevent allergic reactions, doctors should:
  - Avoid giving gemtuzumab ozogamicin to people who are allergic to gemtuzumab ozogamicin or to related substances
  - Give medications to prevent allergic reactions around an hour before each gemtuzumab ozogamicin dose
  - Be particularly careful the first time they give a person gemtuzumab ozogamicin
  - Monitor for symptoms of allergic reactions, such as fever, chills, low blood pressure, increased heart rate, and breathing difficulties.
- If an allergic reaction happens, doctors should:
  - Stop the infusion of gemtuzumab ozogamicin
  - Continue monitoring signs and symptoms of allergic reactions until they disappear
  - Consider giving gemtuzumab ozogamicin again at half the dose, if the reaction was mild or moderate
  - Permanently stop gemtuzumab ozogamicin, if the reaction was severe or life-threatening.

Tumor lysis syndrome

- To prevent tumor lysis syndrome, doctors should:
  - Give people with very high levels of white blood cells treatment to reduce these levels before they receive gemtuzumab ozogamicin
  - Ensure that people drink plenty of water
  - Give medicines to reduce levels of uric acid in the blood, for people who have a high risk of tumor lysis syndrome
  - Monitor for signs and symptoms of tumor lysis syndrome.
- Doctors should treat tumor lysis syndrome according to standard medical practice.

*A side effect is something (expected or unexpected) that you feel was caused by a medicine or treatment you take.

***Supportive care aims to improve a person's condition. For example, for low platelet levels, this includes platelet transfusions and managing any bleeding that may occur.

More results from this article can be found here: **View Scientific Article**

What were the main conclusions from the expert panel?

- People receiving gemtuzumab ozogamicin plus standard chemotherapy have an increased risk of VOD/SOS, compared with people receiving standard chemotherapy alone.
  - The risk of VOD/SOS is greater after a bone marrow transplant.
- However, the risk of VOD/SOS with gemtuzumab ozogamicin therapy may be lower than originally thought. This may be due to improved:
  - Knowledge and management of VOD/SOS
  - Care to prevent and treat VOD/SOS
  - Knowledge on the best dose and schedule to give gemtuzumab ozogamicin.
- This summary provides recommendations for doctors on how to prevent and manage side effects* that can be serious**, including:
  - VOD/SOS
  - Low levels of healthy blood cells
  - Allergic reactions
  - Tumor lysis syndrome.
- These recommendations aim to improve outcomes and quality of life for people with AML.

*A side effect is something (expected or unexpected) that you feel was caused by a medicine or treatment you take.
**A side effect is considered “serious” when it is life-threatening, needs hospital care, or causes lasting problems.

Who sponsored the expert panel?

Pfizer Inc, 235 East 42nd Street, NY, NY 10017. Phone (United States): +1 212-733-2323.

Pfizer funded the expert panel meeting. The doctors were compensated for expenses for their attendance at the expert panel meeting; however, they were not compensated for manuscript or plain language summary preparation.

Pfizer would like to thank all of the people who took part in this review.

Further information

For more information about the recommendations from the expert panel, please visit:

View Scientific Article

For more information on clinical studies in general, please visit:

https://www.clinicaltrials.gov/ct2/about-studies/learn

http://www.cancerresearchuk.org/about-cancer/find-a-clinical-trial/what-clinical-trials-are

Plain language summary writing support was provided by Susan Tan, PhD, of Envision Pharma Group, Inc. and was funded by Pfizer. The authors of the full article were involved in preparing this summary.
